# Supplementary figures and images for: Morphological grounds for the obligate aerial respiration of an aquatic snail: functional and evolutionary perspectives
Source: PeerJ. 2021 Apr 14;9:e10763. doi: 10.7717/peerj.10763 (PMC8052964; doi:10.7717/peerj.10763)

**A**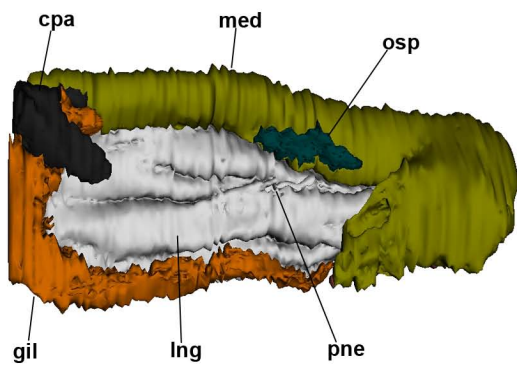**B**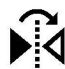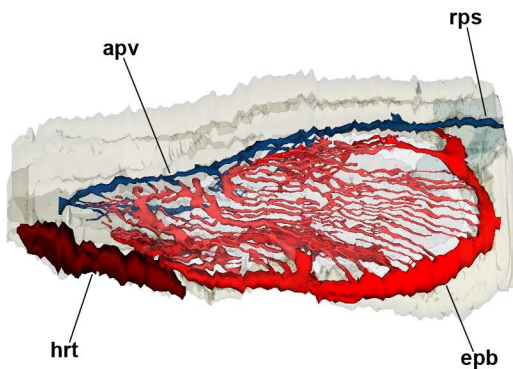**C**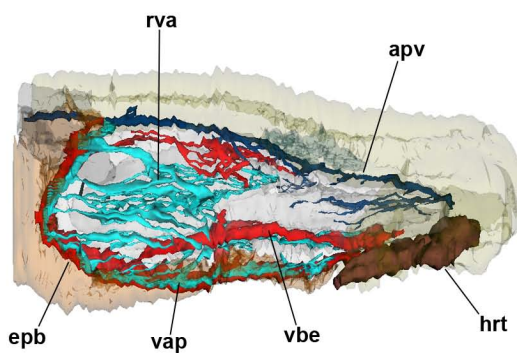**D**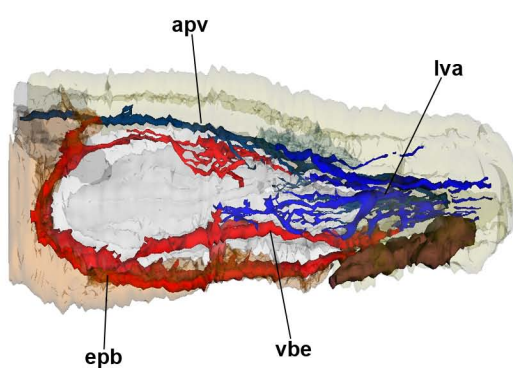**E**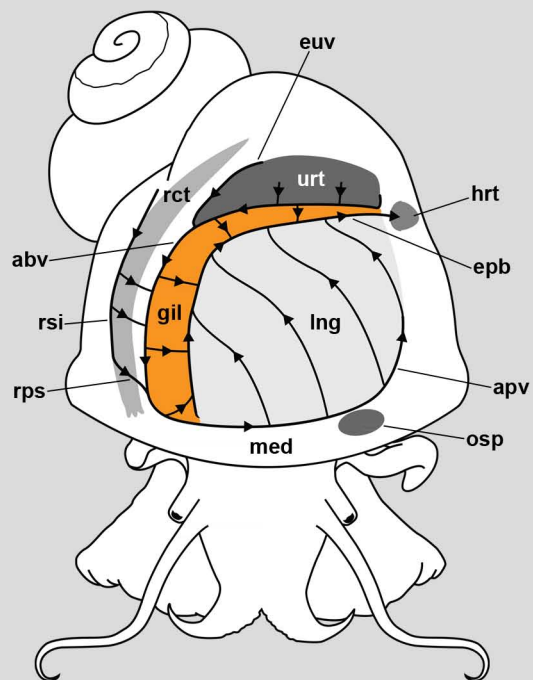**F**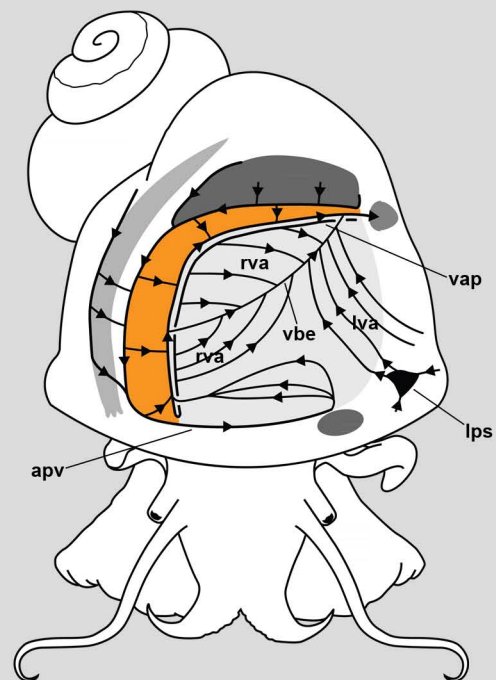

Supplement: Supplemental Information 1 — Left-click on the figure to activate the 3D model using Adobe Acrobat Reader. Left-click and drag to rotate, right-click and drag to zoom-in/out. Structures can be shown/hidden by selecting the pre-defined views from the dropdown menu in the view panel. The 3D PDF was generated using the 3D tool of Adobe Acrobat 9 Pro Extended by importing VRML 2.0 files from Reconstruct v.1.1.0.0. [file peerj-09-10763-s001.pdf]

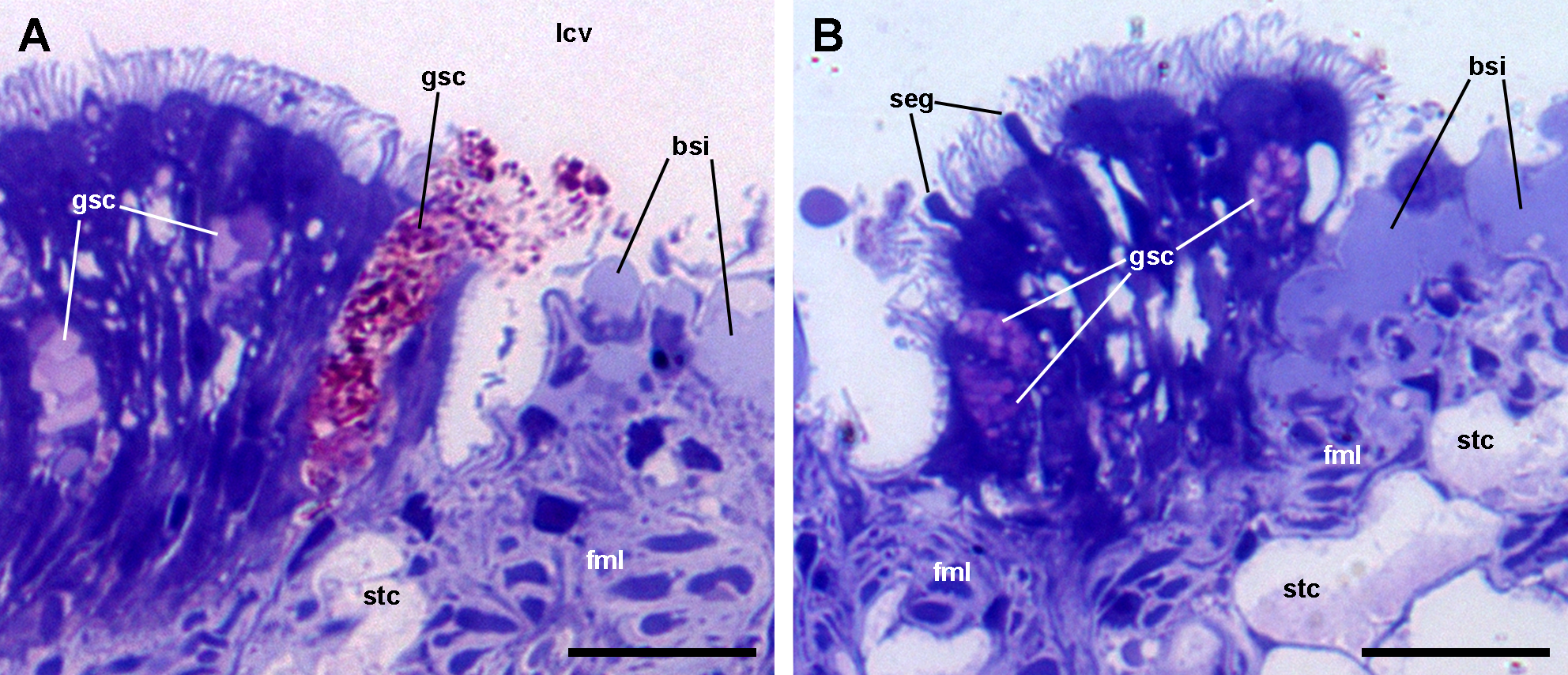

Supplement: Supplemental Information 2 — (A) A ciliary tuft exhibiting granular secretory cells containing orthochromatic granules of varying density and another one with a metachromatic content, which appears being released to the lung cavity. (B) Another ciliary tuft exhibiting granular secretory cells and some apocrine secretory cells exhibiting rather thick protrusions of the apical cytoplasm, which detach as globules toward the pulmonary cavity. In both panels, there are adjacent blood sinuses lying over the fibromuscular layer into which the tufts’ roots penetrate. Abbreviations: bsi, blood sinuses of the respiratory lamina; fml, fibromuscular layer; gsc, granular secretory cells; lcv, lung cavity; seg, secretory globule; stc, storage cells. Toluidine blue. Scale bars represent 20 µm. [file peerj-09-10763-s002.png]
